# Sex Education

**Published:** 1909-03

**Authors:** Follen Cabot

**Affiliations:** New York


### SEX EDUCATION.

BY FOLLEN CABOT, M. D., NEW YORK.

The need of educating the public in matters relating to diseases contracted both through sexual and chance contact, is to my mind a very great one.

The vast amount of ignorance in such matters is appalling and unbelieveable.

The responsibility in the matter rests largely with physicians. We must keep in the van and by preventive measures diminish the ravages of these relentless diseases. What has been done to awaken the minds of people to the dangers and proper care of tuberculosis can also be done in so-called sexual disorders. Here, however, owing to the nature of these diseases, the progress toward enlightenment is necessarily slow. The majority of those afflicted have become so through ignorance. Behind the scene stands the all-powerful sex instinct which often acts regardless of consequences. This must always be so as long as life endures, but I believe by the spread of simple truths, much unnecessary suffering may be prevented.

The entering wedge to the problem, it seems to me, is through early education in matters relating to sex.

The youth of the world must learn about this important part of life, therefore it is better that they should learn the truth in a simple, wholesome way from their parents.

In hospital work during the last ten years, I have been endeavoring to instruct patients about sexual disorders, and by leaflets and other ways have, I think, made some progress. Several other hospitals besides the Post Graduate have taken up the work. We, of course cannot say from year to year how much progress has been made, but I feel sure it is one of the most practical ways to educate the public.

Boards of Health will eventually treat these diseases as they do other contagious diseases. Public opinion will demand it.

The fact that these diseases are spread by chance contact is already, in my opinion, sufficient reason for some such action.

In addition to the leaflets on Gonorrhoea and Syphilis, I have added the following one on the problem of sex and the dangers of ignorance.

DANGER IN IGNORANCE—SOME AXIOMS OF HEALTH.

1.—Sexual relations are not necessary to keep healthy manhood.

2.—If not made use of the power does not become less.

3.—In those men who don't live an active sexual life, seminal emissions (wet dreams) each week or two are natural and can do no harm.

4.—The dangers of serious disease are always great and cannot be avoided outside of marriage.

5.—If disease is contracted it often does permanent harm not only to the man, but to his future wife and children.

6.—A man or woman may be badly diseased and not know it.

7.—To avoid these dangers, physical exercise in a gymnasium and out of doors gives healthy relief.

8.—In boys and young men growth of mind and body progress better without this relation.

9.—To avoid sexual thoughts train the mind by reading and by studying clean books.

10.—Avoid drinking, obscene pictures and vulgar stories. Smoking in moderation is beneficial. Choose companions who respect womanhood.

N. B.—By following the above common sense rules the man will remain strong sexually, keep his body clean and promote his own and other's happiness.

